# 2,2′-(Biphenyl-4,4′-diyldi­oxy)diacetic acid *N*,*N*-dimethyl­formamide solvate

**DOI:** 10.1107/S1600536809025914

**Published:** 2009-07-15

**Authors:** Yu-Juan Cao

**Affiliations:** a School of Chemistry and Environment, South China Normal University, Guangzhou 510006, People’s Republic of China

## Abstract

In the crystal struture of the title compound, C_16_H_14_O_6_·C_3_H_7_NO, the two crystallographically independent benzene rings are coplanar [dihedral angle = 1.00 (2)°]. The crystal structure is stabilized by O—H⋯O hydrogen bonds between the diacid and the solvate dimethylformamide mol­ecule, resulting in the formation of a zigzag chain structure extending parallel to [001].

## Related literature

For general background to biphenyl carbinols and their biological applications, see: Kamoda *et al.* (2006 ▶); Mikami & Yamanaka (2003 ▶); Sallam *et al.* (2006 ▶). For the crystal structures of related compounds, see: Rabnawaz *et al.* (2008 ▶); Tan *et al.* (2005 ▶). For the preparation of the title compound, see: Hayes & Branch (1943 ▶).
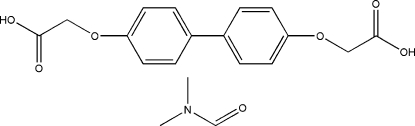


## Experimental

### 

#### Crystal data

C_16_H_14_O_6_·C_3_H_7_NO
*M* *_r_* = 375.37
Orthorhombic, 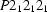

*a* = 7.7471 (15) Å
*b* = 8.1758 (16) Å
*c* = 28.625 (6) Å
*V* = 1813.1 (6) Å^3^
*Z* = 4
Mo *K*α radiation
μ = 0.11 mm^−1^
*T* = 298 K
0.32 × 0.25 × 0.18 mm

#### Data collection

Bruker SMART APEXII CCD area-detector diffractometer
Absorption correction: multi-scan (*SADABS*; Sheldrick, 2004 ▶) *T*_min_ = 0.971, *T*_max_ = 0.984
9412 measured reflections
2079 independent reflections
1778 reflections with *I* > 2σ(*I*)
*R*_int_ = 0.028

#### Refinement

*R*[*F* ^2^ > 2σ(*F* ^2^)] = 0.042
*wR*(*F* ^2^) = 0.116
*S* = 1.05
2079 reflections
246 parameters
H-atom parameters constrained
Δρ_max_ = 0.22 e Å^−3^
Δρ_min_ = −0.19 e Å^−3^

### 

Data collection: *APEX2* (Bruker, 2004 ▶); cell refinement: *SAINT* (Bruker, 2004 ▶); data reduction: *SAINT*; program(s) used to solve structure: *SHELXS97* (Sheldrick, 2008 ▶); program(s) used to refine structure: *SHELXL97* (Sheldrick, 2008 ▶); molecular graphics: *XP* in *SHELXTL* (Sheldrick, 2008 ▶); software used to prepare material for publication: *SHELXTL*.

## Supplementary Material

Crystal structure: contains datablocks I, global. DOI: 10.1107/S1600536809025914/zl2216sup1.cif

Structure factors: contains datablocks I. DOI: 10.1107/S1600536809025914/zl2216Isup2.hkl

Additional supplementary materials:  crystallographic information; 3D view; checkCIF report

## Figures and Tables

**Table 1:** Hydrogen-bond geometry (Å, °)

*D*—H⋯*A*	*D*—H	H⋯*A*	*D*⋯*A*	*D*—H⋯*A*
O6—H6⋯O7^i^	0.82	1.87	2.685 (3)	174
O2—H2⋯O7^ii^	0.82	1.81	2.626 (3)	176
C15—H15a⋯O1^iii^	0.97	2.44	3.149 (2)	129
C17—H17⋯O1^iv^	0.93	2.56	3.248 (3)	131

Symmetry codes: (i) 
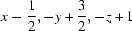
; (ii) 
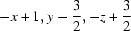
; (iii) 
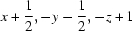
; (iv) 
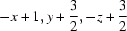
.

**Fig. 1.**
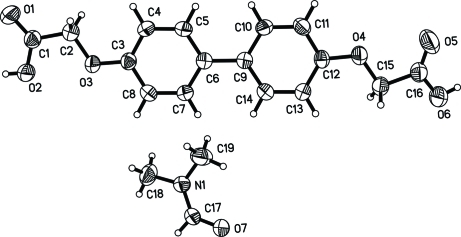
The molecular structure of the title compound with displacement ellipsoids at the 50% probability level. All H atoms are drawn as spheres of arbitrary radius.

**Fig. 2.**
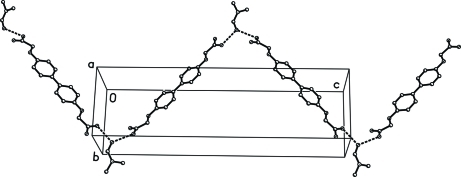
A one-dimensional zigzag chain generated by the hydrogen bonds along the c-axial direction in the title compound. All H atoms are omitted for clarity.

**Fig. 3.**
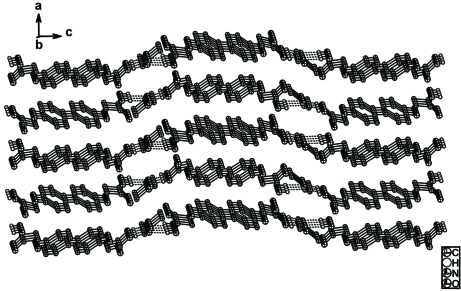
A crystallographic packing diagram of the title compound.

## supplementary crystallographic information

### Comment

Biphenyl carbinols are valuable intermediates in the preparation of new ligands (Mikami *et al.*, 2003; Rabnawaz *et al.*, 2008; Tan *et al.*, 2005) and have shown important biological activities (Kamoda *et al.*, 2006; Sallam *et al.*, 2006). As part of our ongoing study of such biphenyl carbinol compounds, the crystal structure of the title compound is reported in this work.

The molecular structure of the title compound is shown in Fig. 1. The two crystallographically independent benzene rings are coplanar (dihedral angle = 1.00 (2)°) and the two carboxylic acid groups are oriented in different directions. There are no unusual bonds lengths and angles. The C1—O1 and C16—O5 distances in the title compound are 1.197 (3)Å and 1.184 (4)Å, respectively, typical of double bonds.

The –OCH_2_COOH substituents show torsion angles of 176.0 (2)° (C2—O3—C3—C8) and 169.7 (2)° (C15—O4—C12—C11) with respect to the phenyl rings. Intermolecular O—H···O hydrogen bonds between the hydroxyl groups of the diacid and the carbonyl group of the DMF molecule (Table 1) are observed in this structure, thereby forming a one-dimensional zigzag chain structure along the c-axial direction (Fig. 2).

The crystal structure is further stabilized by weak intermolecular hydrogen bonding interactions between the diacids, thus forming a sandwich structure as represented in Fig. 3.

### Experimental

The title compound was prepared according to the general procedure reported by Hayes & Branch (1943). 2-Chloroacetic acid (114 mg, 1.2 mmol) and sodium hydroxide (40 mg, 10 mmol) in 20 ml of *N*,*N*-dimethylformamide (DMF) were stirred for 10 min, followed by addition of 2,2'-dihydroxybiphenyl (186 mg, 1 mmol). The reaction mixture was stirred at 100 °C for 3 h. After cooling, the solution was acidified and extracted with ether. Slow evaporation of ether at room temperature yielded colorless crystals of the title compound. IR(KBr pellet, cm^-1^): 3428.47, 3042.21, 2905.29, 2787.94,1740.59, 1707.78, 1607.65, 1500.03, 1430.90, 1234.94, 830.29, 797.57.

### Refinement

All H atoms were placed in calculated positions and were allowed to ride on their parent atoms; C—H = 0.93 (aromatic C—H), 0.97 (methylene) and 0.96 (methyl) and O—H = 0.82 (hydroxyl) Å; *U*_iso_(H) = 1.2 *U*_eq_ (aromatic and methylene C), *U*_iso_(H) = 1.5 *U*_eq_ (methyl C) and *U*_iso_ (H) = 1.5 *U*_eq_ (O). In the absence of anomalous scatterers and using Mo radiation Friedel pairs were merged prior to refinement.

### Crystal data

**Table:**

C_16_H_14_O_6_·C_3_H_7_NO	*D*_x_ = 1.375 Mg m^−^^3^
*M**_r_* = 375.37	Melting point = 524.9–525.8 K
Orthorhombic, *P*2_1_2_1_2_1_	Mo *K*α radiation, λ = 0.71073 Å
Hall symbol: P 2ac 2ab	Cell parameters from 3913 reflections
*a* = 7.7471 (15) Å	θ = 1.4–27.8°
*b* = 8.1758 (16) Å	µ = 0.11 mm^−^^1^
*c* = 28.625 (6) Å	*T* = 298 K
*V* = 1813.1 (6) Å^3^	Block, colorless
*Z* = 4	0.32 × 0.25 × 0.18 mm
*F*(000) = 792	

### Data collection

**Table:**

Bruker SMART APEXII CCD area-detector diffractometer	2079 independent reflections
Radiation source: fine-focus sealed tube	1778 reflections with *I* > 2σ(*I*)
graphite	*R*_int_ = 0.028
φ and ω scans	θ_max_ = 26.0°, θ_min_ = 1.4°
Absorption correction: multi-scan (*SADABS*; Sheldrick, 2004)	*h* = −9→9
*T*_min_ = 0.971, *T*_max_ = 0.984	*k* = −10→9
9412 measured reflections	*l* = −29→35

### Refinement

**Table:**

Refinement on *F*^2^	Primary atom site location: structure-invariant direct methods
Least-squares matrix: full	Secondary atom site location: difference Fourier map
*R*[*F*^2^ > 2σ(*F*^2^)] = 0.042	Hydrogen site location: inferred from neighbouring sites
*wR*(*F*^2^) = 0.116	H-atom parameters constrained
*S* = 1.05	*w* = 1/[σ^2^(*F*_o_^2^) + (0.0655*P*)^2^ + 0.2914*P*] where *P* = (*F*_o_^2^ + 2*F*_c_^2^)/3
2079 reflections	(Δ/σ)_max_ = 0.001
246 parameters	Δρ_max_ = 0.22 e Å^−^^3^
0 restraints	Δρ_min_ = −0.19 e Å^−^^3^

### Special details

**Table:**

Geometry. All esds (except the esd in the dihedral angle between two l.s. planes) are estimated using the full covariance matrix. The cell esds are taken into account individually in the estimation of esds in distances, angles and torsion angles; correlations between esds in cell parameters are only used when they are defined by crystal symmetry. An approximate (isotropic) treatment of cell esds is used for estimating esds involving l.s. planes.
Refinement. Refinement of F^2^ against ALL reflections. The weighted R-factor wR and goodness of fit S are based on F^2^, conventional R-factors R are based on F, with F set to zero for negative F^2^. The threshold expression of F^2^ > 2sigma(F^2^) is used only for calculating R-factors(gt) etc. and is not relevant to the choice of reflections for refinement. R-factors based on F^2^ are statistically about twice as large as those based on F, and R- factors based on ALL data will be even larger.

### Fractional atomic coordinates and isotropic or equivalent isotropic displacement parameters (Å^2^)

**Table:**

	*x*	*y*	*z*	*U*_iso_*/*U*_eq_	
C1	0.0567 (4)	−0.7098 (3)	0.65314 (10)	0.0437 (7)	
C2	0.0253 (4)	−0.5895 (3)	0.61484 (10)	0.0480 (7)	
H2A	0.0508	−0.6406	0.5850	0.058*	
H2B	−0.0957	−0.5588	0.6148	0.058*	
C3	0.1124 (3)	−0.3291 (3)	0.58665 (9)	0.0385 (6)	
C4	0.0138 (4)	−0.3432 (4)	0.54660 (10)	0.0508 (8)	
H4	−0.0507	−0.4372	0.5411	0.061*	
C5	0.0120 (4)	−0.2161 (4)	0.51477 (10)	0.0506 (8)	
H5	−0.0555	−0.2268	0.4881	0.061*	
C6	0.1058 (3)	−0.0739 (3)	0.52075 (9)	0.0355 (6)	
C7	0.2023 (4)	−0.0632 (3)	0.56174 (9)	0.0415 (6)	
H7	0.2664	0.0308	0.5675	0.050*	
C8	0.2051 (4)	−0.1875 (3)	0.59386 (9)	0.0427 (6)	
H8	0.2705	−0.1762	0.6209	0.051*	
C9	0.1043 (3)	0.0598 (3)	0.48540 (9)	0.0353 (6)	
C10	0.0092 (3)	0.0467 (3)	0.44397 (9)	0.0409 (6)	
H10	−0.0528	−0.0486	0.4383	0.049*	
C11	0.0048 (3)	0.1697 (4)	0.41162 (9)	0.0434 (6)	
H11	−0.0601	0.1567	0.3845	0.052*	
C12	0.0958 (3)	0.3135 (3)	0.41877 (8)	0.0367 (6)	
C13	0.1927 (4)	0.3298 (3)	0.45907 (9)	0.0457 (7)	
H13	0.2558	0.4248	0.4645	0.055*	
C14	0.1950 (4)	0.2043 (3)	0.49115 (10)	0.0481 (7)	
H14	0.2609	0.2173	0.5181	0.058*	
C15	0.1904 (4)	0.5669 (3)	0.38615 (9)	0.0452 (7)	
H15A	0.3090	0.5326	0.3909	0.054*	
H15B	0.1579	0.6398	0.4114	0.054*	
C16	0.1717 (4)	0.6512 (4)	0.33992 (10)	0.0480 (7)	
C17	0.8034 (4)	0.4650 (3)	0.76651 (10)	0.0467 (7)	
H17	0.8715	0.4520	0.7930	0.056*	
C18	0.7724 (5)	0.1739 (4)	0.76798 (13)	0.0648 (9)	
H18A	0.6666	0.1321	0.7807	0.097*	
H18B	0.8570	0.1827	0.7924	0.097*	
H18C	0.8139	0.1009	0.7442	0.097*	
C19	0.6242 (5)	0.3421 (4)	0.70858 (11)	0.0618 (9)	
H19A	0.6722	0.2833	0.6826	0.093*	
H19B	0.6064	0.4543	0.6999	0.093*	
H19C	0.5158	0.2939	0.7172	0.093*	
N1	0.7419 (3)	0.3344 (3)	0.74773 (7)	0.0437 (6)	
O1	−0.0176 (3)	−0.8381 (3)	0.65378 (8)	0.0711 (7)	
O2	0.1679 (3)	−0.6642 (3)	0.68480 (7)	0.0616 (6)	
H2	0.1823	−0.7387	0.7036	0.092*	
O3	0.1272 (3)	−0.4482 (2)	0.61989 (6)	0.0458 (5)	
O4	0.0795 (3)	0.4294 (2)	0.38492 (6)	0.0468 (5)	
O5	0.0680 (5)	0.6151 (4)	0.31136 (10)	0.1098 (12)	
O6	0.2858 (4)	0.7658 (3)	0.33427 (8)	0.0768 (8)	
H6	0.2834	0.7982	0.3072	0.115*	
O7	0.7798 (3)	0.6075 (2)	0.75226 (6)	0.0565 (6)	

### Atomic displacement parameters (Å^2^)

**Table:**

	*U*^11^	*U*^22^	*U*^33^	*U*^12^	*U*^13^	*U*^23^
C1	0.0502 (16)	0.0377 (15)	0.0433 (15)	0.0007 (13)	0.0004 (13)	0.0008 (13)
C2	0.0553 (17)	0.0427 (16)	0.0461 (16)	−0.0061 (14)	−0.0045 (14)	0.0050 (14)
C3	0.0408 (13)	0.0347 (13)	0.0399 (13)	0.0016 (12)	−0.0011 (11)	0.0039 (12)
C4	0.0592 (18)	0.0400 (16)	0.0533 (16)	−0.0170 (15)	−0.0174 (14)	0.0059 (14)
C5	0.0581 (18)	0.0478 (16)	0.0459 (16)	−0.0142 (15)	−0.0207 (14)	0.0058 (14)
C6	0.0337 (12)	0.0357 (13)	0.0372 (13)	0.0027 (11)	0.0011 (11)	−0.0001 (12)
C7	0.0457 (15)	0.0375 (14)	0.0411 (14)	−0.0062 (12)	−0.0066 (12)	0.0010 (12)
C8	0.0468 (15)	0.0463 (15)	0.0350 (13)	−0.0014 (14)	−0.0063 (12)	0.0008 (12)
C9	0.0333 (12)	0.0365 (13)	0.0361 (13)	0.0013 (11)	0.0018 (11)	0.0003 (12)
C10	0.0419 (14)	0.0407 (15)	0.0400 (14)	−0.0089 (13)	−0.0011 (12)	−0.0023 (12)
C11	0.0428 (14)	0.0536 (17)	0.0339 (13)	−0.0037 (14)	−0.0044 (11)	0.0031 (13)
C12	0.0384 (13)	0.0377 (14)	0.0340 (13)	0.0037 (12)	0.0042 (11)	0.0033 (11)
C13	0.0553 (16)	0.0371 (14)	0.0446 (15)	−0.0066 (14)	−0.0071 (13)	0.0052 (13)
C14	0.0561 (16)	0.0479 (17)	0.0403 (15)	−0.0079 (15)	−0.0119 (13)	0.0049 (13)
C15	0.0533 (16)	0.0402 (15)	0.0420 (14)	0.0025 (14)	0.0030 (13)	0.0020 (13)
C16	0.0502 (16)	0.0475 (16)	0.0462 (16)	0.0002 (15)	−0.0003 (14)	0.0065 (14)
C17	0.0606 (18)	0.0434 (16)	0.0360 (14)	0.0003 (15)	−0.0080 (14)	0.0035 (13)
C18	0.082 (2)	0.0402 (17)	0.072 (2)	0.0019 (17)	0.003 (2)	0.0110 (16)
C19	0.068 (2)	0.058 (2)	0.0588 (19)	0.0028 (17)	−0.0166 (16)	−0.0103 (17)
N1	0.0493 (13)	0.0403 (13)	0.0413 (12)	0.0008 (11)	−0.0014 (11)	−0.0021 (10)
O1	0.0894 (17)	0.0499 (13)	0.0740 (15)	−0.0250 (14)	−0.0256 (13)	0.0132 (12)
O2	0.0847 (15)	0.0422 (11)	0.0579 (12)	−0.0134 (12)	−0.0230 (12)	0.0114 (10)
O3	0.0523 (11)	0.0415 (10)	0.0436 (10)	−0.0074 (9)	−0.0085 (9)	0.0107 (9)
O4	0.0498 (11)	0.0469 (11)	0.0436 (10)	−0.0045 (9)	−0.0043 (9)	0.0110 (10)
O5	0.119 (2)	0.123 (3)	0.0881 (19)	−0.058 (2)	−0.0515 (19)	0.060 (2)
O6	0.1004 (19)	0.0726 (16)	0.0573 (14)	−0.0360 (16)	−0.0152 (14)	0.0243 (12)
O7	0.0860 (16)	0.0393 (11)	0.0444 (12)	−0.0036 (11)	−0.0131 (11)	0.0042 (9)

### Geometric parameters (Å, °)

**Table:**

C1—O1	1.197 (3)	C12—O4	1.361 (3)
C1—O2	1.305 (4)	C12—C13	1.383 (4)
C1—C2	1.493 (4)	C13—C14	1.377 (4)
C2—O3	1.407 (3)	C13—H13	0.9300
C2—H2A	0.9700	C14—H14	0.9300
C2—H2B	0.9700	C15—O4	1.415 (3)
C3—O3	1.366 (3)	C15—C16	1.499 (4)
C3—C8	1.377 (4)	C15—H15A	0.9700
C3—C4	1.383 (4)	C15—H15B	0.9700
C4—C5	1.382 (4)	C16—O5	1.184 (4)
C4—H4	0.9300	C16—O6	1.298 (4)
C5—C6	1.382 (4)	C17—O7	1.248 (3)
C5—H5	0.9300	C17—N1	1.287 (4)
C6—C7	1.394 (4)	C17—H17	0.9300
C6—C9	1.489 (4)	C18—N1	1.454 (4)
C7—C8	1.371 (4)	C18—H18A	0.9600
C7—H7	0.9300	C18—H18B	0.9600
C8—H8	0.9300	C18—H18C	0.9600
C9—C14	1.385 (4)	C19—N1	1.446 (4)
C9—C10	1.400 (3)	C19—H19A	0.9600
C10—C11	1.368 (4)	C19—H19B	0.9600
C10—H10	0.9300	C19—H19C	0.9600
C11—C12	1.386 (4)	O2—H2	0.8201
C11—H11	0.9300	O6—H6	0.8200
			
O1—C1—O2	123.9 (3)	C13—C12—C11	118.8 (2)
O1—C1—C2	120.7 (3)	C14—C13—C12	119.4 (3)
O2—C1—C2	115.4 (2)	C14—C13—H13	120.3
O3—C2—C1	112.0 (2)	C12—C13—H13	120.3
O3—C2—H2A	109.2	C13—C14—C9	123.4 (3)
C1—C2—H2A	109.2	C13—C14—H14	118.3
O3—C2—H2B	109.2	C9—C14—H14	118.3
C1—C2—H2B	109.2	O4—C15—C16	106.5 (2)
H2A—C2—H2B	107.9	O4—C15—H15A	110.4
O3—C3—C8	116.8 (2)	C16—C15—H15A	110.4
O3—C3—C4	124.4 (2)	O4—C15—H15B	110.4
C8—C3—C4	118.8 (2)	C16—C15—H15B	110.4
C5—C4—C3	119.3 (3)	H15A—C15—H15B	108.6
C5—C4—H4	120.3	O5—C16—O6	123.8 (3)
C3—C4—H4	120.3	O5—C16—C15	124.1 (3)
C4—C5—C6	123.1 (2)	O6—C16—C15	112.1 (3)
C4—C5—H5	118.5	O7—C17—N1	125.7 (3)
C6—C5—H5	118.5	O7—C17—H17	117.1
C5—C6—C7	116.0 (2)	N1—C17—H17	117.1
C5—C6—C9	121.9 (2)	N1—C18—H18A	109.5
C7—C6—C9	122.0 (2)	N1—C18—H18B	109.5
C8—C7—C6	121.8 (2)	H18A—C18—H18B	109.5
C8—C7—H7	119.1	N1—C18—H18C	109.5
C6—C7—H7	119.1	H18A—C18—H18C	109.5
C7—C8—C3	120.9 (2)	H18B—C18—H18C	109.5
C7—C8—H8	119.5	N1—C19—H19A	109.5
C3—C8—H8	119.5	N1—C19—H19B	109.5
C14—C9—C10	115.7 (2)	H19A—C19—H19B	109.5
C14—C9—C6	122.8 (2)	N1—C19—H19C	109.5
C10—C9—C6	121.6 (2)	H19A—C19—H19C	109.5
C11—C10—C9	122.0 (2)	H19B—C19—H19C	109.5
C11—C10—H10	119.0	C17—N1—C19	121.4 (2)
C9—C10—H10	119.0	C17—N1—C18	121.4 (2)
C10—C11—C12	120.7 (2)	C19—N1—C18	116.8 (3)
C10—C11—H11	119.6	C1—O2—H2	109.4
C12—C11—H11	119.6	C3—O3—C2	117.8 (2)
O4—C12—C13	125.2 (2)	C12—O4—C15	118.6 (2)
O4—C12—C11	116.0 (2)	C16—O6—H6	109.6
			
O1—C1—C2—O3	−178.9 (3)	C9—C10—C11—C12	0.2 (4)
O2—C1—C2—O3	1.4 (4)	C10—C11—C12—O4	−178.6 (2)
O3—C3—C4—C5	−178.3 (3)	C10—C11—C12—C13	0.6 (4)
C8—C3—C4—C5	0.6 (4)	O4—C12—C13—C14	178.4 (3)
C3—C4—C5—C6	0.4 (5)	C11—C12—C13—C14	−0.7 (4)
C4—C5—C6—C7	−1.1 (4)	C12—C13—C14—C9	0.0 (5)
C4—C5—C6—C9	178.8 (3)	C10—C9—C14—C13	0.8 (4)
C5—C6—C7—C8	0.7 (4)	C6—C9—C14—C13	−179.0 (3)
C9—C6—C7—C8	−179.1 (3)	O4—C15—C16—O5	7.5 (5)
C6—C7—C8—C3	0.2 (4)	O4—C15—C16—O6	−170.6 (2)
O3—C3—C8—C7	178.1 (2)	O7—C17—N1—C19	−4.7 (5)
C4—C3—C8—C7	−0.9 (4)	O7—C17—N1—C18	−178.0 (3)
C5—C6—C9—C14	178.9 (3)	C8—C3—O3—C2	176.0 (2)
C7—C6—C9—C14	−1.3 (4)	C4—C3—O3—C2	−5.0 (4)
C5—C6—C9—C10	−0.9 (4)	C1—C2—O3—C3	179.1 (2)
C7—C6—C9—C10	178.9 (2)	C13—C12—O4—C15	11.2 (4)
C14—C9—C10—C11	−0.8 (4)	C11—C12—O4—C15	−169.7 (2)
C6—C9—C10—C11	179.0 (2)	C16—C15—O4—C12	167.4 (2)

### Hydrogen-bond geometry (Å, °)

**Table:**

*D*—H···*A*	*D*—H	H···*A*	*D*···*A*	*D*—H···*A*
O6—H6···O7^i^	0.82	1.87	2.685 (3)	174
O2—H2···O7^ii^	0.82	1.81	2.626 (3)	176
C15—H15a···O1^iii^	0.97	2.44	3.149 (2)	129.0
C17—H17···O1^iv^	0.93	2.56	3.248 (3)	131

Symmetry codes: (i) *x*−1/2, −*y*+3/2, −*z*+1; (ii) −*x*+1, *y*−3/2, −*z*+3/2; (iii) *x*+1/2, −*y*−1/2, −*z*+1; (iv) −*x*+1, *y*+3/2, −*z*+3/2.
